# Mental Health, Cognitive, and Neuropsychiatric Needs in Children and Young People With Wilson Disease

**DOI:** 10.1097/PG9.0000000000000094

**Published:** 2021-07-12

**Authors:** Jemma Day, Marianne Samyn, Sarah Ellen Proctor, Deepak Joshi, Eleanna Pissas, Atchariya Chanpong, Tammy Hedderly, Anil Dhawan

**Affiliations:** From the * Institute of Liver Studies, King’s College Hospital, Denmark Hill, London, United Kingdom; † Paediatric Liver, Gastroenterology and Nutrition, King’s College Hospital, Denmark Hill, London, United Kingdom; ‡ Institute of Liver Studies, King’s College Hospital, Denmark Hill, London, United Kingdom.

**Keywords:** adherence, mental health, neurodevelopment, Wilson disease, cognition

## Abstract

Wilson disease (WD) is associated with neurological, psychiatric, cognitive, and psychosocial difficulties, but there is little data regarding the nature and prevalence of these problems in children and young people (CYP).

**Methods:**

A single-center case-note review to establish the incidence and nature of these issues in CYP with WD, managed before and after multidisciplinary team (MDT) clinics, was established.

**Results:**

Out of 69 (43 males) CYP with WD, 37.8% presented with acute liver failure, 48.6% with chronic liver disease and 13.5% after family screening. Medical treatment was with penicillamine (40), trientene (18), zinc and penicillamine/trientene (11), and zinc monotherapy (2). Twenty-one underwent liver transplantation. After a median follow-up of 9.8 (IQR 6.4–16.9) years, 86% are alive. Six died posttransplantation and 7 grafts were lost. Mental health difficulties were recorded in 49.3%, particularly prevalent in the acute liver failure group (70.8%). Nonadherence was common (50.7%) and associated with greater mental health prevalence. Neurological issues were reported in 36.2% and poor cognition/attainment in 14.5%, consistent across modes of presentation. Four patients had diagnoses of autism spectrum conditions, all diagnosed pre WD. CYP seen within an MDT-clinic had more frequent documentation of all issues examined, but lower levels of late graft loss (94% versus 80%, *P* = 0.07).

**Conclusion:**

Our data highlight the need to offer management in WD patients especially as these aspects are underrecognized in CYP presenting with liver involvement. We aim to highlight the importance of multidisciplinary input when looking after this population beyond transition through to adult services.

## Abstract 2

Supplemental Digital Content is available in the text.

**What Is Known**

- Wilson Disease (WD) is associated with neurological, psychiatric, cognitive and psychosocial difficulties in adults.
- In adults with WD, consistent adherence to treatment is associated with improved outcome and lack of disease progression compared to those classified as nonadherent.

**What Is New**

- Neurological, psychiatric, cognitive, and psychosocial difficulties are highly reported in children with WD with hepatic manifestations.
- Multidisciplinary input is crucial when looking after this population beyond transition through to adult services.

## INTRODUCTION

Neurological symptoms are considered to be rare in children with WD presenting with hepatic onset, reported to affect only 4%–6%.^1–3^ However, there is emerging evidence that neurological symptoms are present and poorly identified or these features may emerge later in children and young people (CYP) diagnosed with hepatic WD.^4–6^ Data regarding the mental health of CYP with WD is also limited to single case studies meaning broader conclusions cannot be drawn.^7–9^ There is some evidence of impaired cognition in CYP with hepatic WD^10,11^ although these impairments are usually subtle. Impairments may manifest as poor school attainment, found in more than a quarter of CYP with WD in one cohort study.^12^ Cognitive impairments have also been reported in adults with WD,^13–15^ and in CYP with other forms of chronic liver disease or portal hypertension^16,17^ highlighting the need to better understand the learning needs of this population.

A successful long-term outcome in WD requires life-long adherence to medication either with chelation therapy or with immunosuppression post liver transplant (LT). In adults with WD, consistent adherence to treatment was associated with improved outcome and lack of disease progression compared to those classified as nonadherent (29.4% and 68.3% versus 2.3% and 45.5%; *P* < 0.001; Masełbas et al^18^). The relationship between nonadherence and significant morbidity and mortality^19^ has been well described in particular during adolescence. Medication nonadherence in CYP with liver disease has been linked to poorer mental health^20^ and to suboptimal physical outcomes (eg, Kerkar et al^21^), highlighting the importance of better understanding in CYP with WD.

## METHODS

A list of all patients diagnosed with WD under the care of King’s College Hospital on record since the database inception in 1981 was generated. Diagnosis of WD was based on presence of liver disease and at least 2 of the following criteria: positive family history, serum caeruloplasmin <0.2 g/L, liver tissue copper >250 mg/g dry weight, presence of Kayser–Fleischer rings, baseline 24-hour urinary copper excretion >1 µmol/24 hours, post Penicillamine 24-hour urinary copper excretion >25 µmol/24 hours, Coombs’ negative hemolytic anemia.^22^ From 2002, genetic testing for WD was routinely undertaken in our center. All patients, including asymptomatic siblings, therefore achieved a score of 4 according to the Leipzig score system. Those diagnosed after 2013 were excluded to allow for sufficient long-term assessment of outcome recording.

The paper and electronic medical notes (electronic patient records; EPR) were carefully reviewed for any documentation of the presence of psychosocial (mental health and neurodevelopmental), neurological (tremor, headache, postural/muscle weakness, neuropathy, and movement disorder) and cognitive (eg, working memory, concentration, attention, and executive functioning) data. A reference to “nonadherence” or “noncompliance” with medication was also recorded. The clinical course, including initial mode of presentation, indication for LT, medication prescribed, where available Magnetic resonance imaging (MRI) data and results of genetic screening was documented, followed by a record of the CYP outcome to date.

Patients were reviewed in a generic pediatric or adult outpatient clinics. Some CYP had been seen within our MDT clinic for young people with liver disease 16–25 years of age and/or a pediatric MDT WD clinic (hepatology and neurology), both established in 2010. Available data regarding psychosocial difficulties as well as data on the neurological and cognitive factors were reviewed and compared between modes of presentation, using descriptive statistics. The primary reasons for referral to the team’s embedded clinical psychologist were then reviewed for WD patients (Supplemental Digital Table 1, http://links.lww.com/PG9/A51). Any description of the factors above was noted across the period of evaluation, and thus difficulties did not pertain to presentation only. It is important to highlight that references in clinical notes to symptoms do not constitute formal clinical diagnoses (eg, of mental health disorders or of specific cognitive profiles). Demographics and clinical data were examined using descriptive statistics and the statistical significance of differences between subgroups were analyzed with nonparametric statistics. The study was approved and performed under institutional ethical guidelines and informed consent was waived (audit number CH057).

## RESULTS

### Medical Data

#### Diagnosis and Presentation

Seventy-four CYP (43 male, 58.1%) fulfilled the diagnostic criteria for WD at median age of 12.28 (IQR 9.70–14.71) years (Table 1). The mode of presentation was acute liver disease/liver failure in 37.8% (n = 28), chronic liver disease (CLD) in 48.6% (n = 36), and following diagnosis of a family member in 13.5% (n = 10). Genetic testing, available after 2002, was performed in 47 patients and confirmed the diagnosis in 42 (homozygous [n = 13], compound heterozygotes [26], single damaging mutation found [n = 3]), and 5 did not screen positively for any of the known mutations.

**TABLE 1.** Demographics, treatment and outcome in children and young people with Wilson disease

N (%)	Total	Acute liver disease/liver failure	Chronic liver disease	Family member of index case
n (Male)	74 (43)	28 (17)	36 (20)	10 (6)
Median age at presentation (years, IQR)	12. 3 (9.70–14.9)	12.3 (9.7–14.7)	11.9 (9.1–13.9)	11.7 (9.1–13.8)
Medication	60 (81%)	14 (50%)	36 (100%)	10 (100%)
Penicillamine	40 (67%)	8	25	7
Trientene	18 (30%)	6	10	2
Zinc monotherapy	2 (3%)	0	1	1
Liver transplantation	21 (28%)	19 (68%)	2 (6%)	0 (0%)
Urgent listing	14 (67%)	14 (74%)	0	0
Median age at liver transplantation (years, IQR)	14.8 (11.9–15.9)	—	—	—
Median duration of follow-up (years, IQR)	9.8 (6.4–16.9)	9.6 (8.3–15.6)	10.3 (6.6–17.5)	10.3 (6.8–16.9)
Multidisciplinary support	34 (46%)	13 (46%)	15 (42%)	6 (60%)

Twenty-four patients (32%) underwent a head MRI. The results were normal in 70.8%. In 5 (20.8%) patients, abnormalities related to WD with copper deposition in the basal ganglia were reported. One was found to have unrelated abnormalities not in keeping with WD. Another patient had resolution of initial MRI features on the second MRI scan, 3 years posttransplant.

#### Treatment

Information on treatment is documented in Table 1. In the acute liver disease/liver failure group, 14 patients were urgently listed for LT and transplanted within 4 weeks of presentation. The other patients (n = 14) were commenced on chelation treatment with Penicillamine, later converted to Trientene in 6. Three failed to respond to medical treatment and were transplanted within 6 months of diagnosis (age 11.3, 11.5, and 13.8 years of age and transplanted 1.9, 5.4, and 2.7 months postdiagnosis, respectively). Two patients required LT after developing decompensated liver disease, 9 and 13 years after diagnosis, respectively.

Those presenting with CLD (n = 36) were managed with chelation treatment with penicillamine (n = 25), trientene (n = 10), and zinc, which was used in combination with penicillamine/trientene in 7 and as monotherapy in 1. Two patients were transplanted after 1.5 and 18.4 years, respectively, due to decompensated liver disease.

Nine family members of index cases (and thus asymptomatic at diagnosis) were treated with chelation therapy (penicillamine n = 7 and trientene n = 2) and 1 with zinc monotherapy. None of the patients in this group required liver transplantation during follow-up.

### Patient Outcome

After median follow-up of 9.8 (IQR 6.4–16.9) years, 64 (86%) CYPs are alive (median age 22.4 [IQR 16.8–29.9] yrs) (Table 1). Ten patients (6 post LT) died at median time of 2.7 (IQR 0.1–11.5) years after diagnosis, with 9 presenting with acute liver disease /liver failure. Four, of which 2 post-LT, died within 2 months of diagnosis; 2 were diagnosed in 1990 before the LT was fully established at our center. Six others (4 post LT) died after median time of 13.1 (range 2.6–22.3) years after diagnosis. Overall LT patient survival (median 13.8 [IQR 11.9–15.2] yrs) was 71% (1-yr 90%, 5-yr 81%, and 10-yr 81%) and graft survival 57% (1-yr 89%, 5-yr 79%, and 10-yr 68%). Six patients (28.6%) lost 7 grafts after a median time of 8 years (range 3 mo–23 yrs). Three (43%) occurred >10 yrs post LT. (Supplemental Digital Info 1, http://links.lww.com/PG9/A52).

### Neurological, Cognitive, Mental Health, and Nonadherence Data

Data on neurological, cognitive, mental health. and nonadherence concerns were documented in 69 patient notes (Table 2).

**TABLE 2.** Neurological, cognitive, mental health, and nonadherence concerns in children and young people diagnosed with Wilson disease

	Neurological concerns (%)	Cognitive concerns (%)	Mental health concerns (%)	Nonadherence (%)
Acute liver disease /liver failure, n = 24	29.2	12.5	70.8	54.2
Chronic liver disease, n = 35	40.0	20.0	40.0	47.0
Family member of index case, n = 10	40.0	0	30.0	55.6
Total, n = 69	36.2	14.5	49.3	50.7

#### Neurological Concerns

Neurological symptoms were reported in 36.2% of cases and were consistent across modes of presentation. Thirteen percent of the 69 case-notes mentioned tremors, 12% mention headaches and 4% referenced muscle weakness. Three percent (2 individuals’ notes) mentioned slurring of speech and motor problems.

#### Cognitive Concerns

The most frequently reported cognitive concerns were memory difficulties. Impaired school performance was described in 14.5% of patients. Concentration difficulties were reported in 4% with no difference between those presenting with acute hepatitis/liver failure compared to the other 2 groups.

1. Mental Health: Mental health concerns (depression, anxiety, rage, and/or psychosis with hallucinations) were referenced in 49.3% and were more common in CYP with acute liver disease/liver failure (70.8%) group compared to the two other groups (40% and 30%, respectively, *P* = 0.028) as shown in Table 2. A concerning deliberate self-harm and suicidal ideation/attempts were noted in 3 (4%) cases. 22% of case-notes record problems with tiredness, fatigue or poor energy.
2. Nonadherence: Nonadherence to medication was mentioned in half (50.7%) of cases. This appeared to be consistent across the modes of presentation (acute hepatitis/liver failure, abnormal liver function tests, and familial screening). Within case-notes that documented nonadherence, mental health problems were mentioned more frequently (70.6%) compared to those who did not document non-adherence (38.2%, *P* < 0.01).
3. Neurodevelopment: Of note, 4 individuals were diagnosed with an autism spectrum condition (ASC) (5.4% of cohort), 3 of whom required LT, and 2 of whom subsequently died <2 years posttransplant.

#### Prevalence of Concerns and Brain MRI Results

MRI brain was not part of the routine management protocol during the period we surveyed (Table 3). Twenty-two had brain MRIs conducted, and results were reported as normal in 17 patients. No difference in prevalence of neurology or cognitive symptoms and/or psychosocial concerns could be identified, comparing patients with normal MRI scans (n = 17: acute hepatitis/liver failure n = 5, chronic liver disease n = 9, family member of index case n = 1) to those with changes associated with WD (n = 5: acute liver disease /liver failure n = 3, chronic liver disease n = 1, family member of index case n = 1). Unsurprisingly, the prevalence of neurological concerns was significantly higher in patients who had an MRI scan (n = 22, 58%) compared to those who did not undergo MRI scanning (n = 47, 28%, *P* = 0.03) with no difference for the other categories.

**TABLE 3.** Comparison of neurocognitive and psychosocial outcomes according to head magnetic resonance imaging

	Neurological concerns (%)	Cognitive concerns (%)	Mental health concerns (%)	Nonadherence (%)
Normal (n = 17)	53	24	65	35
Abnormal (n = 5)	60	20	80	40
Not done (n = 47)	28	11	47	49

### Multidisciplinary Support

Thirty-four (46%) of our cohort were seen in a multidisciplinary (MD) clinic during their follow-up. Eleven patients were seen in the pediatric joint liver-neurology clinic, established in 2009. After 2009, when our MD clinic for young people (age 12–25 years) started, 29 young people were followed up in this clinic as part of their routine follow-up. Mental health concerns, nonadherence, neurological, and cognitive concerns were all documented more frequently in CYP seen in the MD clinic compared to those not seen (non-MD), with no differences in demographics. Although not statistically significant there was a trend for better patient outcome (94% versus 80%) and less late graft loss (>5 years post LT) (11% versus 33%) for those who received MD input compared to the patients who did not.

## DISCUSSION

Neurological, cognitive, mental health, and nonadherence concerns are prevalent in CYP with Wilson disease (WD) during long-term follow-up, and are particularly prevalent in those presenting with acute liver disease/liver failure. The identification of problems was significantly higher in those followed up in MD clinics leading to referral for management, and we would propose that this is likely to lead to a better outcome in terms of liver disease, mental health, neurological management, and we would hope quality of life and participation although this will need further study.

Neurological symptoms were documented in around a third of cases within our clinic population, which is considerably higher than the previous reported prevalence in the literature in CYP. Neurological symptoms were consistently documented across the different modes of presentation rather than only in those with advanced disease or presenting acutely, and were frequently reported in patients with normal MRI scan results. Our data are in keeping with preliminary evidence of subtle neurological manifestations in CYP with hepatic WD.^4^ WD treatment is based on halting (and reversing) the progression of hepatic WD, and studies are needed to determine whether neurological symptoms may develop/progress if there is suboptimal management.

Memory difficulties with specific learning difficulties together with poor school performance were described in 14.5% of patients. Studies have demonstrated impaired cognition in CYP with WD,^10,11^ but such data are scarce. Stock et al^10^ found reduced activity in the anterior cingulate cortex when performing inhibition tasks (an area associated with successful inhibition, a component of executive function). Favre et al^11^ also found relative weaknesses in working memory compared to visuospatial skills in this population, which can impact upon attentional and executive functioning. Cognitive impairments are therefore subtle and may be hard to identify, especially as routine neuropsychological testing is not part of the diagnostic work up or follow-up of CYP with WD.

We had 4 children in our WD clinic reported to have ASCs, and this is higher than expected from population prevalence studies alone (5.4% relative to ~1.04% in the UK general population).^23^ Studies (eg, Bjørklund^24^) have suggested a disturbance in copper (and zinc) metabolism in ASC, suggesting a potential common pathway requiring future systematic investigation.

Mental health concerns were suggested to be present in 49.3% and were particularly common in those presenting with ALF (70.8%). It is not possible from this study to determine whether these concerns relate to the ALF itself or the diagnosis of WD. A report by Rangnekar et al,^25^ exploring quality of life in 282 adults with acute liver failure (28% acetaminophen overdose) found that both those requiring LT (n = 125) and those recovering spontaneously (n = 252) were more likely to report impaired mental health problems compared with the general population (LT group 20%, spontaneous recovery group 31% and general population 10%). For the subgroup aged 18–24 years within this study, impaired mental health was more prevalent in the LT population (35.7%) than the spontaneous recovery group (17.2%; Rangnekar et al^25^) with the reverse relationship found in all other adult age categories. Although references in case-notes clearly do not constitute clinical diagnoses, our data highlight the vulnerability of CYP with WD irrespective of type of presentation.

Our findings are also in keeping with several case reviews in the literature, which typically describe depression and severe behavioral difficulties in CYP subsequently diagnosed with WD.^7–9^ Our findings of mental health problems in those presenting with acute hepatitis/liver failure are in keeping with existing evidence in adults.^26,27^ Mental health concerns were not only documented around the time of diagnosis, but several years later during follow-up. This indicates that these rates do not only reflect untreated WD, or necessarily improve once initiation of treatment has commenced or after liver transplantation. We would recommend that all patients presenting with WD have a history taken that explores mental health problems both before presentation and then during the routine monitoring following diagnosis.

The primary reason for referral to the team’s clinical psychologist is described in Supplemental Digital Table 1, http://links.lww.com/PG9/A51. It is of note that this reflects the primary reason for referral only, and the focus of subsequent therapy may have included other areas; however, the dominant primary reason for referral was around “adjustment to diagnosis,” indicating the challenging nature of this condition.

As is common in adolescents with chronic illnesses, at least half of CYP with WD in our cohort struggle with treatment adherence; nonadherence was mentioned in 50.7% of the case-notes irrespective of the mode of presentation. The case-notes of CYP seen in our MD clinics, in which adherence is routinely assessed were more likely to mention nonadherence compared to those who did not have access to multi-disciplinary assessment/support (62% versus 37%, *P* < 0.01). Our finding that within case-notes that documented non-adherence, mental health problems were mentioned more frequently (70.6%) compared to those who did not document nonadherence (38.2%, *P* < 0.01) is broadly in keeping with existing literature (eg, Burra et al.^28^). This includes findings from our center,^20^ which also indicated a link between non-adherence and poorer mental health in CYP with liver disease.

For those WD patients managed with medical treatment, nonadherence may result in disease progression and decompensation requiring liver transplantation, whereas in the post LT setting nonadherence is associated with graft loss and a higher mortality. Masełbas et al^18^ found that 74% of adults with WD (46.5% with hepatic presentation) reported taking their medications consistently with no regular breaks in their adherence routine. Improvement or stability of the disease was found in 98% of this group compared to 47.8% in those who were nonadherent. Factors influencing adherence were family support and higher education attendance, whereas gender, type of WD, adverse effects of treatment, and duration of the disease did not make a difference (Supplemental Digital Info 2, http://links.lww.com/PG9/A53).

The findings from this retrospective review indicate that psychosocial assessment (such as the HEADSSS psychosocial screening interview)^29^ should be a key component of routine follow up for CYP with WD, particularly given the evidence that CYP with adverse psychosocial circumstances are at a particular risk of poorer physical health outcomes.^30,31^ Psychosocial evaluation should include questions about how the CYP is getting on at school, any concerns regarding learning or attainment, mental health and behavior, as well as the development of referral pathways for patients identified as having concerns and then management in the appropriate services.

Cognitive/full neuropsychological assessments conducted routinely at diagnosis could help to identify impairment patterns which may even be characteristic of other disorders involving the basal ganglia structures, to highlight any learning, mental health, and environmental needs. Of course, documentation in clinical notes does not constitute diagnosis of impairment, and only that such concerns have been documented by the patient, their family or medical team. In the absence of a formal cognitive assessment, CYP with WD (or post-LT for WD) should be asked about their educational progress and attainment, and parent and teachers should be advised to remain vigilant to potential difficulties and adapt educational materials, environment and resources accordingly. Our data would support that a joint Hepatology and Neurological review is optimal for all new patients and MDT follow-up is preferable where possible. Shribman et al^32^ suggested that clinicians must be aware of the “red flags that lie outside their own discipline” when assessing patients with liver disease, a movement disorder or psychiatric illness and have a low threshold for sending through a screen for WD and measuring LFTs. This is particularly true when considering the developing brain of CYP, and any subtle features require specialist evaluation in a timely manner.

Prospective studies are required, in which CYP with WD are formally screened for co-occurring conditions and neuropsychological phenotype at diagnosis and then at regular intervals during follow-up. Effective and timely diagnosis and management of CYP with WD requires a dedicated MD approach involving hepatologists, neurologists and input from psychiatrists, social workers, liaison nurses, and clinical psychologists/neuropsychology to ensure subtle symptoms are identified early and addressed appropriately. This also includes patients who are diagnosed through family screening and are apparently “asymptomatic.”

**TABLE 4.** Comparison of demographics and neuro-cognitive and psychosocial outcomes for children and young people with Wilson disease, seen versus not seen in a multidisciplinary clinic

	Multidisciplinary support	No multidisciplinary support	*P*
N (male)	34 (20)	40 (23)	
Age at diagnosis (median, IQR)	12.5 (10.9–15.1)	12.3 (9.1–14.9)	NS
Duration follow-up (median, IQR)	9.4 (6.6–12.7)	9.8 (6.4–16.9)	NS
Alive at last follow-up	32 (94%)	32 (80%)	0.076
Liver transplantation	9 (26%)	12 (30%)	NS
Retransplantation	2 (22%)	4 (33%)	NS
Nonadherence	62%	37%	0.04
Mental health concerns	68%	31%	0.003
Neurological concerns	53%	20%	0.004
Cognitive concerns	24%	6%	0.036

## Supplementary Material


