# Supplementary material for: Mental Health, Cognitive, and Neuropsychiatric Needs in Children and Young People With Wilson Disease
Source: JPGN Rep. 2021 Jul 12;2(3):e094. doi: 10.1097/PG9.0000000000000094 (PMC10191464; doi:10.1097/PG9.0000000000000094)
Supplement: Supplementary file 1 [file pg9-2-e094-s001.pdf]

Supplementary Table 1: Primary reasons for Referral to Clinical Psychology in Children and Young People with Wilson Disease.

**Reason for referral**	**Number of Referrals**
Adjustment to diagnosis	*7*
Low mood/ depression	*9*
Non-adherence	*7*
Family Work	*3*
Pre-transplant assessment	*2*
Cognitive assessment	*2*
Adjustment to transplant	*2*
Pain management	*2*
Challenging behaviour	*2*
Single referrals for:<br><br>Coping with being on the transplant waiting list<br>Body image- side effects of medication<br>Anger<br>Hearing voices<br>Social difficulties	
