# Supplementary material for: Mental Health, Cognitive, and Neuropsychiatric Needs in Children and Young People With Wilson Disease
Source: JPGN Rep. 2021 Jul 12;2(3):e094. doi: 10.1097/PG9.0000000000000094 (PMC10191464; doi:10.1097/PG9.0000000000000094)
Supplement: Supplementary file 2 [file pg9-2-e094-s002.pdf]

Supplementary Info 1.

Further Information on Patient Outcome- Results

A young man died 6.8 years after diagnosis with gram-negative sepsis whilst being considered for liver transplant. One female responded to chelation treatment after presenting with acute liver failure but died after 13.1 years after developing significant neurological complications. Both had a history of non-adherence to treatment. Of the post liver transplant patients, one patient developed post transplant lymphoproliferative disorder and died 3 years post transplant. The patient who underwent transplant 18 months after presenting with chronic liver disease died of multi-organ failure 2 years post transplant. Two patients, both with complex psychosocial problems and reports of non-adherence passed away 19 and 20 years post liver transplant respectively; one of them 2 years after a third transplant.
