# Supplementary material for: Mental Health, Cognitive, and Neuropsychiatric Needs in Children and Young People With Wilson Disease
Source: JPGN Rep. 2021 Jul 12;2(3):e094. doi: 10.1097/PG9.0000000000000094 (PMC10191464; doi:10.1097/PG9.0000000000000094)
Supplement: Supplementary file 3 [file pg9-2-e094-s003.pdf]

*Supplementary Info 2.*

**Further Information on Patient Outcome- Discussion**

Data on long-term outcome in Wilson disease (especially in children and young people with hepatic involvement) is limited and young patients with Wilson disease are likely to be followed up in a range of different clinical settings (liver transplant clinics, clinics for individuals with chronic liver disease, Wilson disease specific clinics) and in most series they report only on post-transplant outcome. The largest series is from the European Liver Transplant registry, which includes 338 children and young people with Wilson disease aged < 18 years (median age at transplant 14, interquartile range 11.2-16 years) with a median post transplant follow up of 5.4 (interquartile range 1-10.9) years (Pfister et al., 2018). In contrast to our series, only 37% were transplanted for acute liver failure. Patient survival at 1, 5 and 10 years was 87%, 84% and 81% respectively and graft survival 80%, 76% and 71% at the respective intervals. Re-transplantation was required in 14.8%, and therefore less frequent compared to our series (28.9%), which could possibly be explained by the longer follow up in our patients (median 9.4 vs 5.4 years), particularly given 5/7 required re-transplantation more than 5 years after the initial liver transplant. The United Network for Organ Sharing (UNOS) data on 570 patients, including 170 children who underwent liver transplant for Wilson disease, reported better 1-year and 5-year survival in children (90% and 89% respectively) compared to adults (88% and 86% respectively). These figures were 90% and 81% in our children and young people patients, indicating slightly poorer 5 year survival in our cohort. Finally, a large series (n=229) from Austria including adults with hepatic presentation, neurological presentation as well as asymptomatic family members, found that after a median follow up of 14.8 years, 35% remained stable and 15% deteriorated. In comparison to our series, rates of liver transplant (8% vs. 28%) and mortality were lower (7% vs. 14%;) (Bernhardt et al., 2014).

In our cohort the outcome in asymptomatic patients diagnosed from screening because of being family of an index case were reassuring with none of the patients requiring liver transplant during

follow up. However, there was still a high prevalence of neurological concerns (40%), mental health concerns (30%) and non-adherence (55.7%) in this subgroup and this is important to highlight and detect so preventative measures can be considered. Dziezyc et al. (2014) found 15% of 87 pre-symptomatic adults diagnosed as a family member of an index patient and Penicillamine or Zinc, developed neuropsychiatric symptoms during follow up (median follow up time = 12 years). Twenty four percent also developed hepatic dysfunction and there were 5 deaths from hepatic failure. Non-adherence for 3 consecutive months was found in almost half (44.8%) of this group, with 29/37 reporting non-adherence for more than 12 consecutive months and this was associated with poorer outcome.  Combating barriers to effective adherence is therefore of clinical importance, and it is plausible that this presents a cyclical challenge in Wilson disease, given adherence to medication regimens and hospital appointments may be particularly difficult in those hypothesized to have neurological, cognitive, or psychological difficulties. A multi-disciplinary clinic surveillance setting could help with this difficulty.
